# Quality of Life Among Hemodialysis Patients Attending Dialysis Centers in Pokhara Metropolitan, Nepal: A Cross‐Sectional Study

**DOI:** 10.1002/hsr2.71132

**Published:** 2025-07-30

**Authors:** Dhurba Khatri, Nand Ram Gahatraj, Yamuna Chhetri, Bhakta Bahadur KC, Shishir Paudel

**Affiliations:** ^1^ School of Health and Allied Sciences, Faculty of Health Sciences Pokhara University Kaski Nepal; ^2^ Kathmandu Institute of Child Health Hepali Height, Budhanilakantha Kathmandu Nepal; ^3^ National Health Education Information and Communication Centre Teku Nepal

**Keywords:** hemodialysis, kidney failure, quality of life, renal failure

## Abstract

**Background:**

Kidney failure significantly impacts patients' quality of life (QoL), posing a public health concern due to its effects on well‐being, satisfaction, and the increased demand for social and healthcare services. Understanding QoL determinants is critical for improving patient outcomes. Aim. This study assessed the QoL of kidney failure patients undergoing hemodialysis in Pokhara Metropolitan City, Nepal.

**Methods:**

A cross‐sectional study was conducted in 2017 among 132 kidney failure patients attending dialysis centers in Pokhara. Data were collected through face‐to‐face interviews and observations using the Short Form‐36 (SF‐36) Health Survey and a structured checklist. Descriptive statistics were used for frequency distribution, percentage, mean score, and standard deviation. Student's unpaired *t*‐test and ANOVA were used to compare the mean score differences of the quality of life among the groups.

**Results:**

The mean overall QoL score was 38.9 ± 6.21, with physical component summary (PCS) and mental component summary (MCS) scores of 36.55 ± 6.74 and 41.23 ± 13.36, respectively. While most participants (86.5%) were satisfied with personal relationships, 10.6% reported experiencing social discrimination or stigma, and 55% had not been invited to social events. Economic status and participation in social organizations were significantly associated with higher QoL scores across PCS, MCS, and overall QoL (*p* < 0.05).

**Conclusion:**

The majority of patients suffering from kidney failure had overall poor quality of life. Social support and wealth index played a significant role in the quality of life. The findings suggest the need of enhancing social support systems and promoting vocational rehabilitation could improve QoL for this population.

## 1 Introduction

Chronic kidney disease (CKD) refers to an enduring impairment in kidney structure or function, lasting for a period exceeding 3 months [1]. This condition primarily arises due to chronic conditions such as diabetes and hypertension, which are leading global health challenges [2]. The rapid rise in these risk factors has made CKD a major global health burden, contributing to increased prevalence, substantial healthcare costs, and poor patient outcomes [3, 4]. Globally, CKD affects approximately 9.46% of the population [5]. In 2017, CKD caused approximately 1.2 million deaths, while the use of dialysis accounted for 0.041% of the population. Between 1990 and 2017, the all‐age prevalence of CKD rose by 29.3%, reaching 697.5 million cases in 2019 [6, 7].

The Global Burden of Disease Study reported 1.4 million CKD‐related deaths in 2019, reflecting a 20% increase from 2010 and positioning CKD among the leading causes of mortality worldwide [7]. In Asia, CKD prevalence doubled between 1990 and 2019, increasing from 202.4 million to 431.2 million cases [8]. In Nepal, CKD affects 6.0% of the population, with a higher prevalence among males (6.5%) compared to females (5.7%). The prevalence notably increases with age, reaching 11.5% among individuals aged 60 years and above, and 2.6% among those aged 20–39 years [9]. CKD in its early stages is associated with numerous adverse outcomes, including progressive kidney function loss, cardiovascular disease (CVD), diabetes mellitus, elevated cholesterol levels, increased waist‐to‐hip ratios, premature death, and diminished quality of life (QoL) [9, 10]. It significantly limits patients' cognitive, physical, and social functioning, negatively impacting their overall physical and mental well‐being [11].

QoL refers to an individual's perception of their life, considering cultural factors, personal goals, and societal expectations. Health‐Related QOL (HRQOL) specifically evaluates physical, mental, and social domains of health influenced by personal experiences and beliefs [12]. In recent years, QoL has become a crucial outcome measure in medicine, providing insights into how health conditions affect individuals beyond clinical indicators [13, 14].

In Nepal, previous studies have reported significantly lower QoL in hemodialysis patients compared to kidney transplant recipients. A cross‐sectional study from Kathmandu found that hemodialysis patients had significantly lower scores in physical (*p* < 0.001), psychological (*p* < 0.001), social relationship (*p* = 0.012), and environmental health (*p* = 0.004) domains [15]. Similarly, a study from Biratnagar reported an mean QoL score of 48.9 ± 13.7 among hemodialysis patients [16]. Another study from Bir Hospital found that the prevalence of good QoL among hemodialysis patients was 53.64% in the physical component summary (PCS), 22.05% in the mental component summary (MCS), and 21.28% in the kidney disease component summary (KDCS), compared to 13.19% in patients without hemodialysis [17].

The first kidney transplantation in Nepal was performed in 2008, following legislation passed in 2000 [18, 19]. Subsequently, the government introduced free dialysis in 2013 and free kidney transplantation services in 2017 [18]. Despite these initiatives, the treatment of CKD, especially end‐stage renal disease (ESRD), remains costly and largely unaffordable for most Nepalese citizens [20, 21]. Even with government assistance covering dialysis costs, patients still face significant out‐of‐pocket expenses for medications, transportation, and dietary requirements [15]. The Government of Nepal pays NRs 2500 to hospitals per dialysis, providing a significant amount of financial support [20]. However, this subsidy does not cover the cost of essential medications, leaving patients with a significant financial burden [20, 22].

Given the high burden of CKD and its profound impact on physical and mental well‐being, a comprehensive assessment of QoL among hemodialysis patients in Nepal is crucial. While prior studies have examined QoL, they have primarily focused on overall scores, with limited exploration of clinical, socioeconomic, and psychosocial determinants. Moreover, most research has been concentrated in Kathmandu and Biratnagar, overlooking other urban regions such as Pokhara. This study aims to evaluate QoL among kidney failure patients attending dialysis centers in Pokhara Metropolitan City, examining the multidimensional influences of clinical, economic, and psychosocial factors. By addressing these gaps, the findings will provide valuable insights to guide healthcare policies and interventions aimed at improving patient outcomes.

## 2 Methodology

### 2.1 Study Design and Study Setting

A descriptive cross‐sectional study was conducted among hemodialysis patients attending all five dialysis centers in Pokhara Metropolitan, Nepal, from September to October 2017. Geographically, Pokhara Metropolitan is the largest metropolitan city in Nepal and was selected as the study area as it attracts diverse population from the western Nepal seeking tertiary care. The five dialysis centers collectively had the capacity to serve up to 175 patients per week.

### 2.2 Sample Size and Study Population

**Figure 1:**
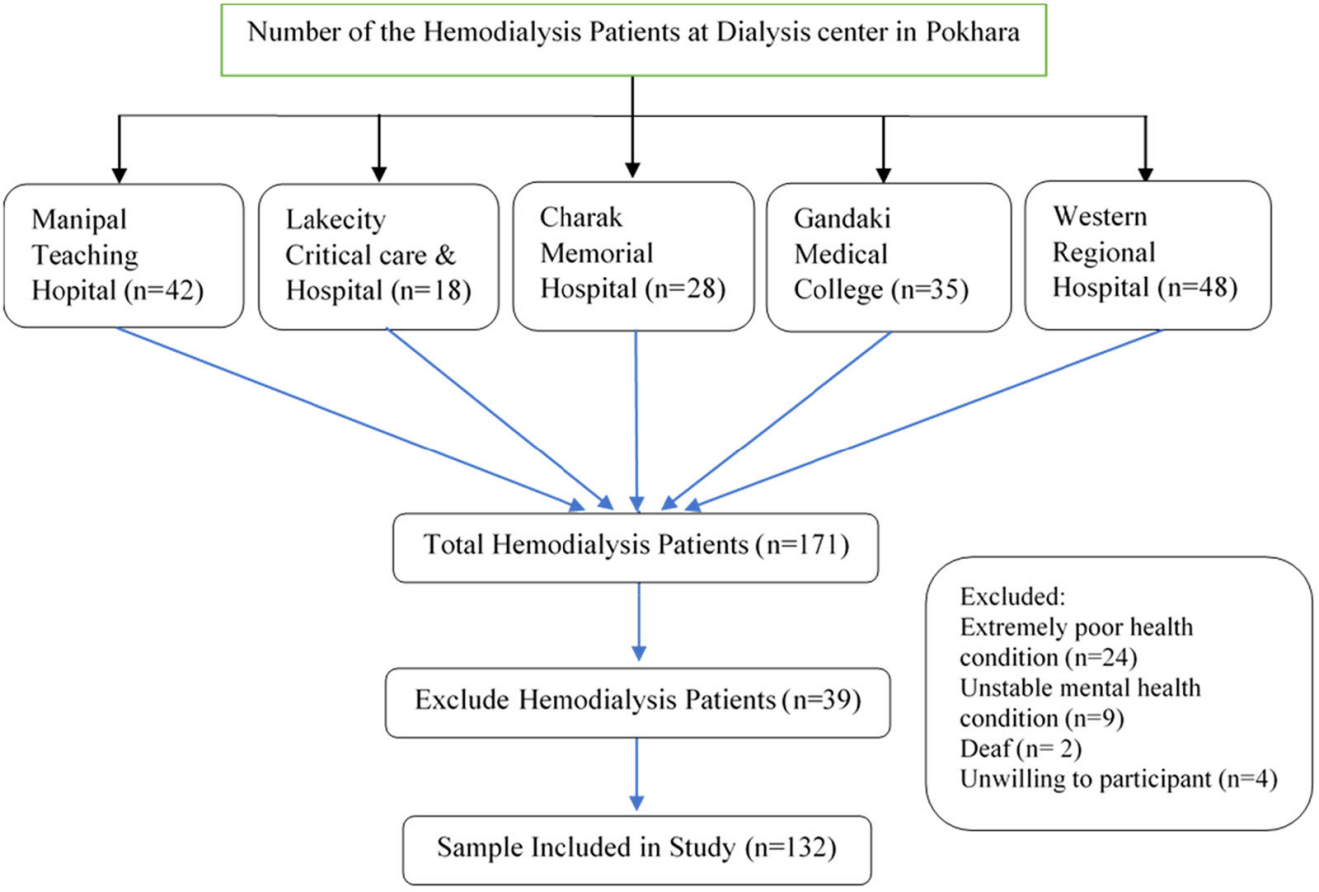
Overview of Participant Enrollment and Exclusion.

The study employed a census method, including 132 out of 171 eligible patients aged 18 years or older who were undergoing maintenance hemodialysis (Figure 1). A total of 39 patients were excluded: 16 due to severe illness that prevented meaningful participation, 12 because of hearing or speech impairments that precluded effective communication, 7 due to cognitive impairments, and 4 who declined to participate. Thus, the study population comprised all eligible hemodialysis patients from the dialysis centers in Pokhara Metropolitan City. While no exclusions were made based on age, sex, ethnicity, or religion, individuals who could not engage in a valid interview due to clinical or communication‐related barriers were excluded for methodological consistency.

### 2.3 Data Collection Instruments

The data were collected using a face‐to‐face interview using a predefined interview schedule. To maintain consistency across the interview process in five different hospitals, all interviews were conducted by two designated researchers (D.K. and Y.C.). One researcher interviewed male participants, while the other interviewed female participants to ensure participant comfort and ease. This approach helped standardize the interview process and maintain uniformity across all study sites.

The data questionnaire consisted of four parts: Part I covered socio‐demographic information, Part II assessed social well‐being, and Part III evaluated quality of life using the Short Form Health Survey (SF‐36) [23]. Short Form health survey (SF‐36) questionnaires consist of eight domains: physical functioning, social functioning, role emotional, role, physical pain, general health, vitality, and mental health for measuring the quality of life [23]. The PCS and MCS scores derived from the SF‐36 Health Survey [24]. The each domains' scores of short form‐36 range from 0 to 100, with 0 representing no health‐related quality of life and 100 describing the perfect health‐related quality of life.

The interview schedule was developed based on a literature review to gather comprehensive information about patients' backgrounds and social well‐being. It was pretested among 12 dialysis patients from Rupandehi to ensure clarity and reliability. All the interviews were conducted at the hospital while participants waited for their turn for dialysis. To maintain privacy and confidentiality, the interviews were held in a private space. Each interview lasted approximately 20–30 min and was conducted only after obtaining informed consent from the participants.

### 2.4 Statistical Analysis

Raw data was cleaned, coded, and entered using Raw data were cleaned, coded, and entered using EpiData version 3.1 before being transferred to the Statistical Package for Social Sciences version 20 for analysis. QoL scores were calculated according to the SF‐36 manual [23]. Descriptive statistics were used for frequency distribution, percentage, mean score, and standard deviation. Inferential statistics, including Student's independent *t*‐test and ANOVA, were employed to compare mean QoL scores among different groups.

### 2.5 Ethical Consideration

Ethical clearance was obtained from the Institutional Review Committee of Pokhara University (Ref No.27/074/75). The permission was taken from the District Public Health Office (DPHO), Kaski, to conduct the study in their catchment area. Written informed consent was obtained from all participants, who were assured of their right to withdraw from the study at any time. Counseling was provided to participants with poor QoL at the end of the study.

## 3 Results

### 3.1 Socio‐Demographic Characteristics

Among the 132 participants, males accounted for nearly two‐thirds (73.5%). The mean age was 48.60 ± 14.90 years, ranging from 18 to 83 years. Regarding ethnicity, 43.2% were relatively advantaged Janajatis, followed by the upper caste group (33.3%) and Dalits (18.9%). Most of the participants (87.1%) were married. Only 11.4% were illiterate, and the rest were literate. Nearly half (44.7%) were in their first year of dialysis (Table 1).

**Table 1:** Socio‐demographic characteristic of participants.

Socio‐demographic characteristic	Total participants (*n* = 132)	
	Frequency (*n*)	Percentage (%)
Sex of participants		
Male	97	73.5
Female	35	26.5
Age		
≤ 40	45	34.1
41–50	23	17.4
51–60	30	22.7
≥ 60	34	25.8
Mean age ± Standard deviation	48.60 ± 14.898	
Ethnicity		
Dalit	25	18.9
Disadvantage Janajatis	4	3.0
Religious Minorities	2	1.5
Relatively advantage Janajatis	57	43.2
Upper caste group	44	33.3
Religion		
Hindu	91	68.9
Buddhist	29	22.0
Christian	10	7.6
Muslim	2	1.5
Marital status		
Unmarried	10	7.6
Married	115	87.1
Widow/Divorce	7	5.3
Education Status		
Illiterate	15	11.4
Informal	26	19.7
Basic	29	22.0
Secondary	51	38.6
University degree or more	11	8.3
Employment Status		
Unemployed	11	8.3
Employed	121	91.7
Disability		
Yes	89	67.4
No	43	32.6
Dialysis Duration		
≤ 1 Year	59	44.7
1–2 Years	33	25.5
2–3 Years	20	15.2
Above 3 Years	20	15.2
Mean duration ± Standard deviation	2.022 ± 1.85 years, Min 18 Days, Max 8 Years	
Economic Status		
Lowest quintile (16–38)	24	18.2
Second quintile (39–45)	10	7.6
Third quintile (46–56)	24	18.2
Fourth quintile (57–70)	31	23.5
Fifth quintile (71–100)	43	32.6

### 3.2 Social Wellbeing Related Characteristics

Most (86.3%) were satisfied with their family relationships, while 68.2% expressed satisfaction with the support from friends. Only 10.6% faced discrimination, primarily at a moderate level (78.6%). Stigma was reported by 9.1%, and 44.7% were invited to participate in social activities, of whom 38.6% accepted. Additionally, 24.2% of participants were involved in social organizations (Table 2).

**Table 2:** Social wellbeing related characteristic.

Social wellbeing related variable	Frequency (*n*)	Percentage (%)
Satisfied with a personal relationship (*n* = 132)		
Satisfied	114	86.5
Neutral	8	6.2
Dissatisfied	10	7.7
Satisfied with support get from friends		
Satisfied	90	68.2
Neutral	13	9.1
Dissatisfied	30	22.7
**Experienced being discriminated**		
Yes	14	10.6
No	118	89.4
Level of discrimination (*n*) = 14		
Mild	1	7.1
Moderate	11	78.6
Severe	2	14.3
**Experienced stigma**		
Yes	12	9.1
No	120	90.9
Invited to participate in social activities		
Yes	59	44.7
No	73	55.3
Participated in social activities		
Yes	51	38.6
No	81	61.4
Involve in social organization		
Yes	32	24.2
No	100	75.8

### 3.3 Descriptive Statistics of Quality of Life

**Figure 2:**
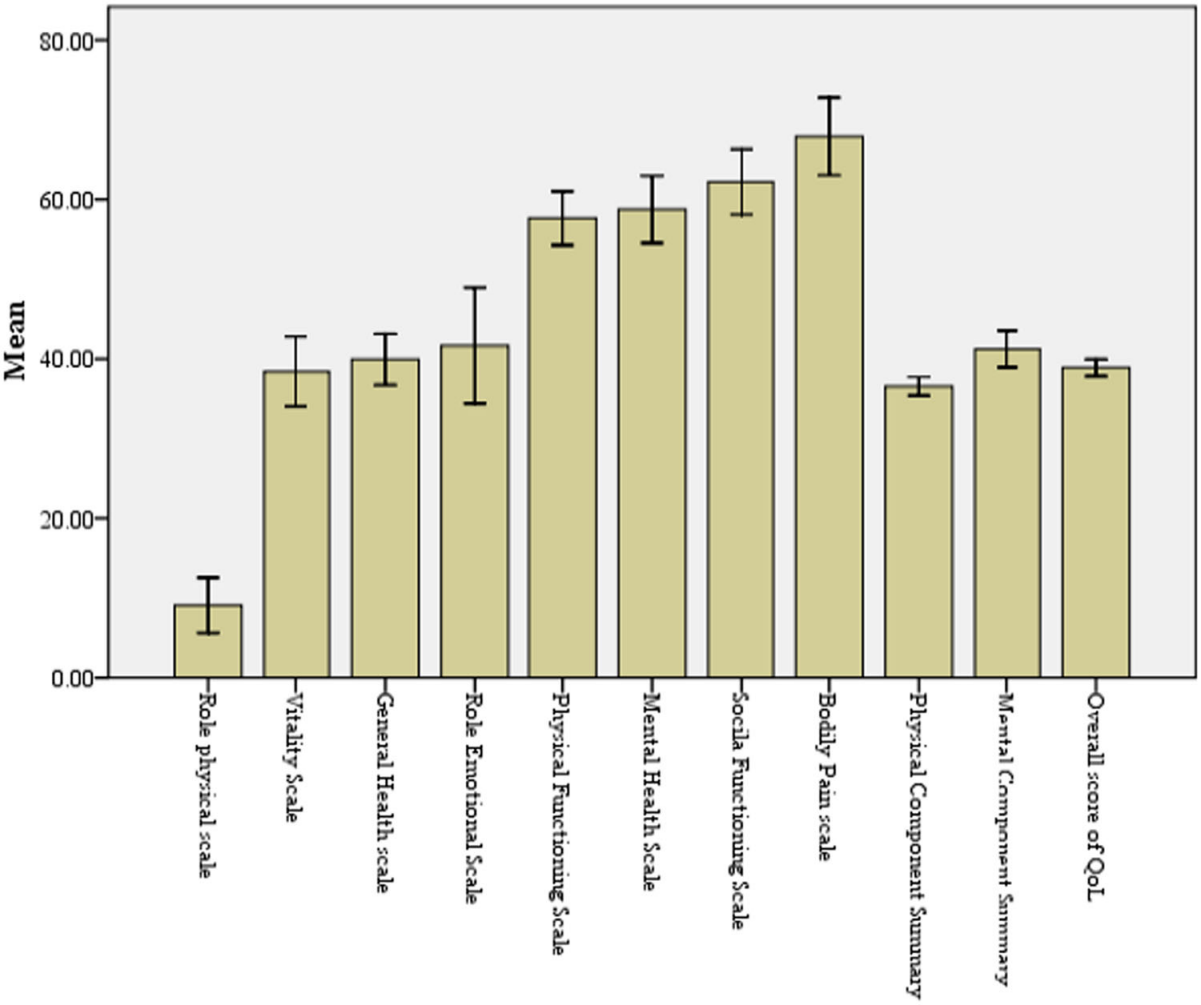
Mean score with standard error bars.

The highest‐scoring domain of the SF‐36 quality‐of‐life measure was bodily pain (67.91 ± 28.33), while the role‐physical domain had the lowest score (9.09 ± 20.8). The PCS mean score was 36.55 ± 6.74, lower than the MCS mean score of 41.23 ± 13.36. The overall quality‐of‐life score averaged 38.88 ± 6.20 (Figure 2).

### 3.4 Comparative Statistical Analysis of the Mean Scores

Comparative analysis of categorical variables revealed significant associations between PCS, MCS, and overall quality‐of‐life scores and certain variables. Participants with higher economic status, involvement in social activities, and participation in social organizations showed statistically significant mean differences across these dimensions (*p* < 0.05). Education status also played a significant role, with illiterate participants scoring significantly lower on the MCS (*p* = 0.010) (Table 3).

**Table 3:** Comparative statistical analysis of the mean scores.

Variables	PCS	*p* value	MCS	*p* value	Overall	*p* value
Sex						
Male	36.21 ± 7.12	0.117	41.77 ± 13.41	0.827	38.98 ± 6.04	0.389
Female	37.49 ± 5.56		39.73 ± 13.33		38.61 ± 6.13	
Age						
≤ 40	35.21 ± 6.82	0.084	40.97 ± 13.34	0.025*	38.09 ± 6.09	0.106
41–50	35.56 ± 6.45		47.55 ± 9.11		41.55 ± 5.53	
51–60	36.57 ± 6.82		41.91 ± 12.62		39.24 ± 5.19	
≥ 60	38.96 ± 6.38		36.67 ± 15.04		37.81 ± 7.22	
Ethnicity						
Dalit	38.43 ± 7.00	0.224	39.60 ± 15.74	0.587	39.02 ± 7.22	0.782
Disadvantage Janajatis	36.48 ± 3.92		37.15 ± 12.21		36.82 ± 5.05	
Religious minorities	42.89 ± 2.75		41.11 ± 2.06		42.00 ± 2.40	
Relatively advantage Janajatis	36.60 ± 6.53		40.16 ± 13.21		38.38 ± 6.19	
Upper caste group	35.12 ± 6.96		43.90 ± 12.48		39.51 ± 5.89	
Religion						
Hindu	36.62 ± 6.91	0.547	41.15 ± 13.89	0.998	38.89 ± 6.42	0.879
Buddhist	35.79 ± 6.04		41.18 ± 13.84		38.49 ± 6.27	
Christian	36.75 ± 7.65		42.02 ± 8.37		39.39 ± 4.69	
Muslim	42.89 ± 2.75		41.11 ± 2.06		42.00 ± 2.40	
Marital status						
Unmarried	36.74 ± 6.98	0.140	41.39 ± 13.45	0.664	39.06 ± 6.23	0.684
Married	32.78 ± 2.79		42.34 ± 13.45		37.56 ± 5.76	
Widow/Divorce	38.72 ± 5.15		36.87 ± 15.96		37.80 ± 6.97	
Education status						
Illiterate	37.96 ± 4.13	0.028*	33.53 ± 13.49	0.010*	35.75 ± 6.84	0.055
Informal	39.75 ± 7.61		37.12 ± 14.60		38.44 ± 7.13	
Basic	34.45 ± 7.31		40.72 ± 13.39		37.59 ± 5.20	
Secondary	36.17 ± 6.17		44.54 ± 11.43		40.35 ± 5.54	
University degree	34.29 ± 6.19		47.33 ± 12.84		40.81 ± 6.86	
Employment status						
Employed	32.31 ± 4.79	0.029*	53.41 ± 10.52	0.001*	42.86 ± 6.07	0.026*
Unemployed	36.93 ± 6.78		40.11 ± 13.07		38.52 ± 6.11	
Disability						
Yes	37.24 ± 6.40	0.089	39.47 ± 12.64	0.030*	38.35 ± 6.14	0.160
No	35.11 ± 7.28		44.85 ± 14.22		39.98 ± 6.27	
Dialysis duration						
≤ 1 Year	38.23 ± 6.79	0.072	39.90 ± 13.30	0.627	39.07 ± 5.73	0.693
1–2 Years	34.99 ± 6.66		42.11 ± 14.57		38.55 ± 6.93	
2–3 Years	35.81 ± 6.25		44.22 ± 12.91		40.01 ± 6.80	
> 3 Years	34.86 ± 6.49		40.65 ± 12.23		37.75 ± 5.92	
Economic status						
First quintile (16–38)	40.42 ± 5.67	0.002*	31.40 ± 12.37	< 0.001**	35.91 ± 6.43	0.027*
Second quintile (39–45)	37.51 ± 4.19		44.28 ± 9.69		40.89 ± 4.51	
Third quintile (46–56)	37.50 ± 4.97		37.24 ± 12.88		37.37 ± 5.62	
Fourth quintile (57–70)	36.44 ± 8.37		44.27 ± 12.57		40.36 ± 6.58	
Fifth quintile (71–100)	33.70 ± 6.27		46.01 ± 12.34		39.86 ± 5.91	
Experienced being discriminated						
Yes	35.16 ± 3.22	0.005*	32.90 ± 13.19	0.601	34.03 ± 5.91	0.620
No	36.71 ± 7.04		42.21 ± 13.09		39.46 ± 6.01	
Experienced stigma						
Yes	36.29 ± 3.86	0.891	28.03 ± 8.68	< 0.001**	32.16 ± 4.04	0.103
No	36.57 ± 6.98		42.54 ± 13.05		39.56 ± 5.99	
Invited to participate in social activities						
Yes	34.81 ± 7.41	0.008*	47.40 ± 10.84	< 0.001**	32.16 ± 4.04	< 0.001**
No	37.94 ± 5.84		36.22 ± 13.17		39.56 ± 5.99	
Participated in social activity						
Yes	34.77 ± 7.46	0.016*	47.63 ± 11.07	< 0.001**	41.20 ± 5.93	0.001*
No	37.66 ± 6.04		37.19 ± 13.15		37.42 ± 5.95	
Involve in social organization						
Yes	33.81 ± 6.54	0.008*	47.97 ± 10.19	0.001*	40.89 ± 5.90	0.035*
No	37.42 ± 6.60		39.06 ± 13.57		38.24 ± 6.19	

* Signification at < 0.05.

** Signification at < 0.001.

## 4 Discussion

This study provides crucial insights into the QoL among hemodialysis patients in Nepal, highlighting the interplay between clinical, economic, and psychosocial factors. The mean duration of hemodialysis was 2.02 years, which slightly contradicts the findings under a similar setting from Dharan, where the average time of dialysis was reported to be 2.5 months [25]. The increase in the mean duration might be attributed to the government's provision of free dialysis services, which has likely improved treatment adherence and retention over time [26]. Additionally, advances in dialysis technology, improved patient management strategies, and increased awareness about chronic kidney disease (CKD) may have contributed to longer treatment durations [27].

Across the eight domains of QoL, the highest score was observed in the pain domain, followed by social functioning and mental health. Likewise, the lower score was observed in the domain related to physical functioning followed by vitality. The greater impairment of the PCS compared to the MCS suggests that fatigue and mobility limitations significantly impact daily life. These findings are consistent with the studies from Nepal [17, 28] Saudi Arabia [29], and North America [30], where the physical domain was most affected. However, other studies from Pokhara and Chitwan Nepal, reported greater impairment in the mental domain [31, 32]. Similarly, in South Kerala, India, the physical component had the lowest mean score (38.11 ± 20.87), while the mental component was higher (47.33 ± 16.88), indicating moderate mental well‐being [33]. A study from Bangladesh also showed more pronounced mental health issues, with PCS and MCS scores of 37.19 ± 8.1 and 45.94 ± 7.5, respectively [34]. Findings from Sri Lanka further corroborate this contrast, with PCS and MCS scores of 35.5 ± 15.0 and 39.6 ± 12.3 [35]. These variations may arise from differences in psychological support systems, cultural attitudes toward illness, and family/community support. Age was significantly associated with MCS, consistent with past findings from Nepal [17, 25, 31] and other regions, including Ethiopia, and Saudi Arabia [4, 36, 37]. Studies from Kerala, India, and Bangladesh reported that younger patients had better mental‐health‐related QoL, while age did not significantly affect PCS [33, 34]. Older patients may experience greater psychological distress due to increased responsibilities, financial burdens, and family concerns, as observed in Palestine [38], Oman [39], India [40]. Additionally, cognitive decline and reduced social engagement may further contribute to mental health challenges among the elderly.

Educational status was significantly associated with both PCS and MCS, supporting findings from previous studies from Nepal such as from Pokhara [31] and Chitwan [32] as well as studies from Thailand, and Ethiopia [4, 41]. Higher education may facilitate better understanding of the disease, improved adherence to prescribed treatments, and more effective utilization of healthcare resources, ultimately enhancing QoL [27]. Socioeconomic status plays a crucial role in shaping health literacy, empowering patients to actively manage their condition and seek necessary support [42]. Economic status was significantly associated with PCS, MCS, and overall QoL which is consistent with the other studies conducted in similar setting [4, 41, 43]. However, a longitudinal study did not find such association [44]. Financial disparities significantly influence access to nutrition, medications, and mental health support [45]. In Ethiopia, higher family income (β 15.33; 95% CI: 11.33–19.33, *p* < 0.001) and higher educational status (β 7.9; 95% CI: 4.10–11.66, *p* < 0.001) predicted better PCS, while higher family income (β 10.10; 95% CI: 5.10–15.10, *p* < 0.001) was a predictor of better MCS [4]. Similarly study from Sri Lanka further highlight that financial hardship is a key determinant of QoL among dialysis patients [35].

Employment status was significantly associated with MCS, consistent with findings from previous studies from Nepal [17, 32], but differing from findings observed in Thailand and Ethiopia [4, 31, 41]. Employment provides financial stability and a sense of purpose, enhancing mental well‐being, while unemployment may lead to stress, social isolation, and diminished self‐worth. Evidence from Southeast Asian nations further corroborates that employment status significantly influences mental health outcomes among hemodialysis patients [46]. However, in contrast to expectations, employed participants did not show significantly higher PCS scores in our study. One possible explanation is that many patients in Nepal continue to work out of financial necessity despite declining physical health, which may contribute to increased physical fatigue, stress, and reduced physical quality of life. In such cases, employment may be more burdensome than beneficial to physical health.

Social engagement factors, such as participation in social organizations, invitations to social activities, experience of discrimination or stigma were significantly associated with QoL (PCS, MCS, and overall scores). This underscores the crucial role of social interactions in enhancing well‐being, fostering emotional resilience, reducing isolation, and improving coping mechanisms [47, 48]. Study from China reinforce the importance of social support in improving treatment adherence and psychological outcomes [49]. Furthermore, discrimination by society had a statistically significant association with PCS, consistent with past studies [50]. Discrimination may negatively affect physical health, including reducing access to healthcare and limiting healthy behaviors. In comparison with the mental component score, there were differences on the mean score of stigma face which was supported by previous study [49, 51, 52]. It may be due to stigma lead to diminished treatment adherence in patients with chronic kidney disease. social acceptance, being able to perform social responsibilities and self‐esteem which are directly linked to the employment status, income and education status of an individual [35, 46]. However, contrary to expectations, participants who reported being socially active or involved in social organizations did not have significantly higher PCS scores. One possible explanation is that social participation in this context may not require a high level of physical functioning. Individuals may still attend social events or remain socially involved despite experiencing physical limitations, due to cultural norms or social obligations. Additionally, social engagement may be driven more by emotional or psychological needs than by physical capability. These findings highlight the complex interplay between social roles and physical health in this population and warrant further context‐specific investigation.

Despite being few of the studies assessing the quality of life among Hemodialysis patients in Nepal, it has some limitations. First, the SF‐36 questionnaire has not yet been formally validated in the Nepalese population; as a result, domain scores should be interpreted with caution. The future research should focus on psychometric validation of this instrument. Second, as a hospital‐based study, the findings may not be generalizable to all hemodialysis patients in Nepal due to potential selection bias. Third, due to time constraints we were unable to collect detailed clinical information, such as diabetes status, hemoglobin levels, and albumin levels, which have been shown in previous studies to be associated with QoL. We recognize this as a limitation and suggest that future studies incorporate these clinical factors to offer a more comprehensive understanding of their impact on QoL. Additionally, reliance on self‐reported data for quality of life and socioeconomic characteristics may introduce reporting bias. To address these limitations, future research should consider employing larger, more diverse samples and longitudinal study designs to track changes in QoL over time. Furthermore, mixed‐method approaches, including qualitative perspectives, could provide richer insights into patient experiences and the socio‐cultural factors influencing well‐being.

## 5 Conclusion

The present study showed evidence that patients with end‐stage kidney failure on hemodialysis had poor QoL. Gender, ethnicity, employment status, education level, and religion had no statistical significance, whereas economic status & social well‐being‐related factors were statistically substantial. Efforts should be made to enhance social support mechanisms and provide vocational rehabilitation opportunities for these individuals. By addressing the social discrimination and stigma faced by kidney failure patients, it is possible to enhance their overall well‐being and satisfaction with life. Furthermore, research studies based on qualitative methods & longitudinal studies to explore the determinants of quality of life among chronic kidney disease patients are recommended with better sampling procedures.

## Author Contributions

**Dhurba Khatri:** conceptualization, methodology, data curation, formal analysis, visualization, writing – original draft, writing – review and editing, investigation, project administration, validation, resources. **Nand Ram Gahatraj:** conceptualization, supervision, visualization, writing – review and editing, validation, methodology. **Yamuna Chhetri:** methodology, data curation, investigation, formal analysis, project administration, writing – review and editing. **Bhakta Bahadur KC:** visualization, writing – review and editing, formal analysis. **Shishir Paudel:** writing – review and editing, writing – original draft, visualization, formal analysis.

## Conflicts of Interest

The authors declare no conflicts of interest.

## Transparency Statement

The lead author Dhurba Khatri affirms that this manuscript is an honest, accurate, and transparent account of the study being reported; that no important aspects of the study have been omitted; and that any discrepancies from the study as planned (and, if relevant, registered) have been explained.

## Data Availability

The data that support the findings of this study are available from the corresponding author upon reasonable request.
